# Genome-Wide Identification and Characterization of *WRKY* Gene Family in Peanut

**DOI:** 10.3389/fpls.2016.00534

**Published:** 2016-04-26

**Authors:** Hui Song, Pengfei Wang, Jer-Young Lin, Chuanzhi Zhao, Yuping Bi, Xingjun Wang

**Affiliations:** ^1^Shandong Provincial Key Laboratory of Crop Genetic Improvement, Ecology and Physiology, Biotechnology Research Center, Shandong Academy of Agricultural SciencesJinan, China; ^2^Department of Molecular, Cell, and Developmental Biology, University of California, Los AngelesLos Angeles, CA, USA

**Keywords:** *Arachis*, disease resistant, gene duplication, phylogenetic relationship, WRKY

## Abstract

WRKY, an important transcription factor family, is widely distributed in the plant kingdom. Many reports focused on analysis of phylogenetic relationship and biological function of WRKY protein at the whole genome level in different plant species. However, little is known about WRKY proteins in the genome of *Arachis* species and their response to salicylic acid (SA) and jasmonic acid (JA) treatment. In this study, we identified 77 and 75 WRKY proteins from the two wild ancestral diploid genomes of cultivated tetraploid peanut, *Arachis duranensis* and *Arachis ipaënsis*, using bioinformatics approaches. Most peanut WRKY coding genes were located on *A. duranensis* chromosome A6 and *A. ipaënsis* chromosome B3, while the least number of *WRKY* genes was found in chromosome 9. The *WRKY* orthologous gene pairs in *A. duranensis* and *A. ipaënsis* chromosomes were highly syntenic. Our analysis indicated that segmental duplication events played a major role in *AdWRKY* and *AiWRKY* genes, and strong purifying selection was observed in gene duplication pairs. Furthermore, we translate the knowledge gained from the genome-wide analysis result of wild ancestral peanut to cultivated peanut to reveal that gene activities of specific cultivated peanut *WRKY* gene were changed due to SA and JA treatment. Peanut *WRKY7, 8* and *13* genes were down-regulated, whereas *WRKY1* and *12* genes were up-regulated with SA and JA treatment. These results could provide valuable information for peanut improvement.

## Introduction

WRKY transcription factors, a large family of regulatory proteins, are widely distributed in plant and non-plant species (Eulgem et al., 2000; Riechmann et al., 2000; Zhang and Wang, 2005; Rushton et al., 2010; Rinerson et al., 2015). WRKY proteins are characterized by the WRKY domain, which includes about 60 amino acids with a conserved WRKYGQK heptapeptide (Eulgem et al., 2000; Rushton et al., 2010). WRKY proteins contain one or two WRKY domains and either one of two types of zinc finger motif at C-terminal (Eulgem et al., 2000; Rushton et al., 2010). WRKY proteins could be classified into three groups (I, II, and III) based on the number of WRKY domains and the type of the zinc finger motifs. Group I WRKY proteins include two WRKY domains and a zinc-finger motif (Eulgem et al., 2000; Rushton et al., 2010). Each group II and III WRKY proteins contain a single WRKY domain, and a CX_4−5_CX_22−23_HXH and CX_7_CX_23_HXC zinc-finger motifs in C-terminal region, respectively (Eulgem et al., 2000; Rushton et al., 2010). Group II is further subdivided into five subgroups (IIa-IIe) based on phylogenetic relationship (Eulgem et al., 2000).

By binding to W-box *cis*-element (C/TTGACT/C) in target gene promoter, WRKY proteins are involved in a variety of biological functions, including different plant developmental programs, as well as diverse abiotic and biotic stress response (Eulgem et al., 2000; Rushton et al., 2010). WRKY proteins were implicated to modulate plant development, such as, trichome morphogenesis (Johnson et al., 2002), flowering (Luo et al., 2013), seed development (Luo et al., 2005), dormancy and germination (Zhang et al., 2004; Zentella et al., 2007; Zou et al., 2008), and senescence (Robatzek and Somssich, 2002). Recent studies revealed that WRKY proteins were involved in response to abiotic stresses (Rushton et al., 2012), such as salt (Wu et al., 2009), drought (Ren et al., 2010; Jiang et al., 2012), cold (Zou et al., 2010), and wounding (Cheong et al., 2002). For instance, expression of *AtWRKY46* gene could significantly induced by drought, H_2_O_2_ and salt stress, and *wrky46* mutant in Arabidopsis was more sensitive to salt and osmotic stress compared to control (Ding et al., 2014). Expression of *TaWRKY44* gene in tobacco could improve drought, salt and osmotic stress tolerance (Wang et al., 2015). Previous studies indicated that WRKY proteins play crucial roles in pathogen defense (Eulgem and Somssich, 2007; Rushton et al., 2010) and insect (Grunewald et al., 2008; Skibbe et al., 2008). Xu et al. (2006) found that expression of *AtWRKY18* increased resistance to *Pseudomonas syringae*, but its co-expression with *AtWRKY40* or *AtWRKY60* made plants more susceptible to both *P. syringae* and *Botrytis cinerea*. Furthermore, increasing studies documented that WRKY proteins are involved in defense-relative hormone signal transduction, salicilic acid (SA) and jasmonic acid (JA)-mediated defenses, where SA triggered defenses against biotrophic pathogens and JA participates in the response to necrotrophic pathogens (Li et al., 2004; Schluttenhofer et al., 2014). For example, in Arabidopsis, *AtWRKY50* and *AtWRKY51* promoted SA biosynthesis (Gao et al., 2011); *AtWRKY17* and *AtWRKY33* genes were induced after JA treatment (Journot-Catalino et al., 2006). Overexpression of *AtWRKY28* and *AtWRKY46* genes could promote the expression of ICS1 and PBS3 genes through SA signaling pathway (van Verk et al., 2011). Additionally, recent study showed that the expression of 12 *WRKY* genes from Catharanthus roseus responded to JA (Schluttenhofer et al., 2014), and that the expression of 49 Salvia miltiorrhiza *WRKY* genes was signification up- or down-regulated by JA treatment (Li et al., 2015).

WRKY proteins were studied extensively in a variety of plant species (Eulgem et al., 2000; Wu et al., 2005; Wei et al., 2012a; Liu et al., 2014; Song and Nan, 2014; Song et al., 2014, 2016). However, current basic knowledge of WRKY proteins and the characterization of specific WRKY proteins involved in disease resistance from species in genus *Arachis* are still limited. Peanut (*Arachis hypogaea* L.) is an important oil crop grown throughout the tropics and subtropics regions. Especially in Asia, which accounts for 64% of the world yield, peanut provides a similar amount of calories from soybean (Bertioli et al., 2011). To date, 80 species in genus *Arachis* were identified and classified into nine taxonomic sections (Bertioli et al., 2011). Wild species are diploid, but cultivated peanut is allotetraploid (AABB). The wild ancestral species of cultivated peanut are generally considered to be *Arachis duranensis* and *Arachis ipaënsis*, which contributed the A and B sub-genomes, based on morphology, cytology, fertility of the interspecific hybrid and molecular studies (Kochert et al., 1996; Seijo et al., 2004, 2007; Ramos et al., 2006). Plant diseases have been a major reason for peanut yield losses. Interestingly, the disease resistant capacity of wild peanut was proved much higher than that of cultivated peanut (Herbert and Stalker, 1981; Pande and Narayana Rao, 2001; Simpson, 2001). Therefore, disease resistant genes from wild type species could be valuable resources for cultivated peanut improvement. Recently, the whole genome sequences of *A. duranensis* and *A. ipaënsis* have been released (Bertioli et al., 2016), which provided an important resource for genome wide analysis of the disease resistant genes. To fill the knowledge gap, we identified 75 AdWRKY and 77 AiWRKY proteins from *A. duranensis* (Aradu.V14167.a1.M1) and *A. ipaënsis* (Araip.K30076.a1.M1), respectively, by bioinformatics approaches, and then we studied the phylogenetic relationship, the genome-wide distribution pattern, gene duplication event, and selection pressure of *WRKY* genes in the two species. Additionally, we deduced potential disease-resistant *Arachis* WRKY proteins based on the functional study of *Arabidopsis* WRKY (AtWRKY) proteins and then we transferred the knowledge to cultivated peanut to determine the expression pattern of *WRKY* genes by quantitative real-time RT-PCR (qRT-PCR) in different tissues, and we monitored the *WRKY* transcriptional changes with SA and JA treatment to preliminarily validate the disease-resistant potential. Our results provide a comprehensive genome-wide knowledge of WRKY proteins in the two wild ancestral species of peanut and a preliminary knowledge of specific WRKY proteins potentially involved in disease resistance in peanut.

## Materials and methods

### *WRKY* proteins in *A. duranensis* and *A. ipaënsis* genomes

The *A. duranensis* (Aradu.V14167.a1.M1) and *A. ipaënsis* (Araip.K30076.a1.M1) genome sequences were downloaded from http://peanutbase.org/ (Bertioli et al., 2016). The Hidden Markov Model (HMM) profile of the WRKY domain (PF03106) was downloaded from the pfam database (http://pfam.janelia.org). It was used to match each WRKY protein in genomes using HMMER program (Finn et al., 2011). To verify the reliability of results, all protein sequences were checked in the pfam database. The AtWRKY protein sequences were downloaded from Arabidopsis Information Resource website (TAIR, http://www.arabidopsis.org). WRKY sequences of *Glycine max* (GmWRKY), *Lotus japonicus* (LjWRKY) and *Medicago truncatula* (MtWRKY) were obtained from previous studies (Yin et al., 2013; Bencke-Malato et al., 2014; Song et al., 2014; Song and Nan, 2014).

### Classification of *A. duranensis* and *A. ipaënsis WRKY* proteins

The AtWRKY, AdWRKY, and AiWRKY domains were extracted based on pfam database. Multiple sequences alignment was executed by MAFFT 7.0 program (Katoh and Standley, 2013). AtWRKY domains were used as query to categorize the AdWRKY and AiWRKY proteins based on the phylogenetic tree described by previous study (Song et al., 2014). The phylogenetic trees were constructed using MEGA 6.0 (Tamura et al., 2013) using neighbor-joining model with 1000 replicates. Other phylogenetic trees were inferred according to the parameters.

### The genome-wide distribution pattern, gene duplication event and selection pressure of *WRKY* genes

The chromosomal location information of AdWRKY and AiWRKY proteins was obtained from peanutbase website (http://peanutbase.org/). The map was generated using MapInspect software (http://mapinspect.software.informer.com/).

Different types of gene duplication existed in genome, while we focused only on tandem and segmental duplication events in this study. To identify gene duplication events in *AdWRKY* and *AiWRKY* genes, duplicated *GmWRKY* genes were used as query to construct phylogenetic trees of *AdWRKY* and *AiWRKY* genes, respectively. Colinearity between *Arachis* and *Glycine* is more conserved (Nagy et al., 2012). If *GmWRKY* and *AdWRKY* or *AiWRKY* genes were clustered in pairs in phylogenetic tree, the gene pairs were considered as orthologous genes (Dutilh et al., 2007; Altenhoff and Dessimoz, 2012). We classified gene duplication events of *AdWRKY* and *AiWRKY* based on the information from the duplicated *GmWRKY* (Yin et al., 2013). Moreover, we identified orthologous genes between *AdWRKY* and *AiWRKY* based on phylogenetic relationship using above method (Dutilh et al., 2007; Altenhoff and Dessimoz, 2012).

Non-synonymous (Ka) and synonymous (Ks) substitution of each duplicated *AdWRKY* and *AiWRKY* genes were calculated by PAL2NAL program (Suyama et al., 2006), which is based on codon model program in PAML (Yang, 2007). Generally, Ka/Ks (ω) = 1, >1, and < 1 indicated neutral, positive, and purifying selection, respectively. To detect whether the *AdWRKY* and *AiWRKY* genes underwent positive selection under site model and branch-site model, PAML program was applied in this study. In site model, M0 (one ratio), M1a (neutral), M2a (selection), M3 (discrete), M7 (beta), and M8 (beta + ω) were applied to selection pressure analysis. We detected absolute value in the ω ratio parameter among sites using likelihood ratio test (LRT) for M1a vs. M2a, M0 vs. M3 and M7 vs. M8. In Branch-site model, the ω ratio between clades was used for comparison. The phylogenetic trees were constructed using sequences from the amino acid by the MEGA 6.0 (Tamura et al., 2013). Posterior probabilities were estimated using the Bayes Empirical Bayes (BEB) method (Yang, 2007).

### Plant materials and hormone treatment

To analyze expression pattern of deduced potential SA- and JA- related *WRKY* genes in different tissues, root, stem, leaf, flower, and seed of cultivated peanut (Luhua 14) were harvested from experimental farm in September, 2015. To examine the expression of 13 deduced potential SA- and JA- related *WRKY* genes under SA and MeJA treatment, peanut seeds were germinated on humid filter paper in growth chamber at 28°C, and then growth for 4 weeks at room temperature (~32°C). SA and MeJA solution (0.1 mM) was applied to the leaves, respectively. Fresh leaves were harvested after 0, 6, 24, 36, and 48 h of treatments.

### Gene expression analysis by qRT-PCR

Total RNA was extracted using CTAB method (Chang and Puryear, 1993). The first-strand cDNAs were obtained using 2 μg of DNA-free RNA using Reverse Transcriptase M-MLV System (Takara, Dalian, China).

Actin gene was used as a reference gene to quantitative the expression of *AhWRKY* genes (Xia et al., 2013). The reaction was carried out using fluorescent dye SYBR-Green (Takara, Dalian, China). qRT-PCR was carried out using Fast Start Universal SYBR Green Master (ROX) with a 7500 real-time PCR machine (ABI). The reaction was carried out as follows: 30 s at 95°C for denaturation, followed by 40 cycles of 5 s at 95°C and 30 s at 60°C. A melting curve analysis was performed at the end of the PCR run over a range of 55–99°C. Three biological replicates were used. The ΔΔCt method was used for quantification (Livak and Schmittgen, 2001). A pairwise student's *t*-test was performed to obtain the *P* values using JMP 9.0. If *P* < 0.05, we considered the *WRKY* genes as differential expressed genes.

## Results and discussion

### *WRKY* proteins in two wild type peanuts

A total of 75 and 77 WRKY proteins were identified from *A. duranensis* and *A. ipaënsis*, respectively, using bioinformatics approach (Tables S1, S2). They were named as AdWRKY1 to AdWRKY75, and AiWRKY1 to AiWRKY 77, respectively. Among 75 AdWRKY sequences, six were partially sequenced without complete sequence in the released genome draft, namely AdWRKY3, 11, 15, 24, 55, and 73. The length of other 69 full-length sequences ranged from 291 to 3477 bp (Table S1). AdWRKY8 and 35 sequences contained internal termination codon, indicating these two sequences were pseudogene or sequencing errors. In 77 AiWRKY sequences, nine were partially sequenced without complete sequence in the genome draft, including AiWRKY10, 24, 29, 30, 36, 40, 69, 70, and 71. The length of remaining 68 AiWRKY sequences ranged from 291 to 3564 bp (Table S2).

AdWRKY proteins could be classified into three groups, group I (16 sequences), group II (46 sequences) and group III (13 sequences). Similarly, AiWRKY proteins could be classified into group I (14 sequences), group II (48 sequences), and group III (15 sequences) based on the number of WRKY domain and the type of zinc finger motif (Table 1). Forty-six group II AdWRKY proteins could further be classified into five subgroups, IIa (4 sequences), IIb (10 sequences), IIc (18 sequences), IId (7 sequences), and IIe (7 sequences); The 48 group II AiWRKY could be classified into subgroup IIa (4 sequences), IIb (10 sequences), IIc (18 sequences), IId (7 sequences), and IIe (9 sequences) based on the phylogenetic trees (Table 1, Figures S1, S2). The number and type of AdWRKY and AiWRKY proteins in the corresponding subgroup was similar (Table 1). These results were consistent with the results described by Ding et al. (2015) that no significant gene domain or number difference was detected between two *Gossypium* species. Similar results were found in *Brassica* species (Table 1).

**Table 1:** **Number of WRKY proteins in plants**.

**Species**	**Group I**	**Subgroup IIa**	**Subgroup IIb**	**Subgroup IIc**	**Subgroup IId**	**Subgroup IIe**	**Group III**	**Total**
*Arachis duranensis*	16	4	10	18	7	7	13	75
*Arachis ipaensis*	14	4	10	18	7	9	15	77
*Gossypium raimondii*^a^	20	7	16	26	16	13	14	112
*Gossypium arboreum*^a^	19	7	16	26	14	13	14	109
*Gossypium hirsutum*^b^	16	7	12	28	15	11	10	99
*Brassica rape*^c^	31	7	17	38	14	13	25	145
*Brassica rapa* ssp. *pekinensis*^d^	31	7	17	38	14	13	25	145
*Brassica oleracea* var. *capitata*^e^	36	6	17	36	14	15	24	148

a Data from Ding et al. (2015);

b data from Dou et al. (2014);

c data from Kayum et al. (2015);

d data from Tang et al. (2014);

e *data from Yao et al. (2015)*.

Previous studies found that WRKYGQK heptapeptide prone to mutate (Dou et al., 2014; Song and Nan, 2014; Song et al., 2014; Ding et al., 2015). WRKYGQK sequence is considered to be important for recognizing and binding to W-box elements. WRKYGKK sequences represented the major variant in AdWRKY and AiWRKY proteins. We found that WRKYGKK was also the most common variant in LjWRKY (Song et al., 2014) and GmWRKY (Song et al., 2016). The WRKYGKK sequence in tobacco WRKY12 bound specifically to WK-box (TTTTCCAC), which was significantly different from the consensus sequence of a W-box (C/TTGACT/C) (van Verk et al., 2008). WRKYGKK sequence was observed in AdWRKY21, 25, 26, 30, 59, 63, 69, and 72 proteins (Table S1). Among them, AdWRKY21, 25, 30, 59, 69, and 72 proteins belonged to subgroup IIc; AdWRKY26 belong to subgroup IId, and WRKYGKK sequence was located in the N-terminal WRKY domain sequence of AdWRKY63 protein, which belonged to group I (Table S1). Three WRKYGEK sequences were found in AdWRKY18 (subgroup IIc), AdWRKY20 (group III), and AdWRKY62 (group I). GRKYGEK, WRKYDEK, WRKYEEN, and WRKYGRK heptapeptides were detected in AdWRKY51 (subgroup IId), AdWRKY37 (group III), AdWRKY7 (subgroup IIc) and C-terminal WRKY domain sequence of AdWRKY63 (group I) (Table S1). In AiWRKY proteins, five WRKYGKK peptides were found in AiWRKY14, 18, 30, 32, and 48, which belonged to subgroup IIc (Table S2). Two WRKYGEK sequences were found in AiWRKY24 (group III) and AiWRKY26 (subgroup IIc) proteins, and two WRKYEEN sequences were identified in AiWRKY59 (subgroup IIc) and AiWRKY77 (subgroup IIc) proteins. Moreover, RKKYGQK, WCKYGEK, and WRKHGQK sequences were found in AiWRKY76 (group III), AiWRKY71 (subgroup IIc) and AiWRKY25 (group III) (Table S2). Group I AiWRKY contained one WRKYDKK and one WHKYGKK in WRKY N-terminal domain. These results showed that WRKYGQK sequence in subgroup IIc WRKY proteins prone to mutate. In soybean WRKY proteins we also found that some WRKY domains in subgroup IIc were likely to mutate (Song et al., 2016). These results suggested that subgroup IIc proteins might potentially carry out a variety of biological functions. It is noteworthy that mutation occurred in WRKYGQK sequence of AdWRKY63 and AiWRKY51, suggesting their possible involvement in multiple biological functions when bind to different W-box elements.

WRKY proteins contained two types of zinc-finger motif, CX_4−5_CX_22−23_HXH and CX_7_CX_23_HXC (Eulgem et al., 2000; Rushton et al., 2010). We found these two zinc-finger motifs were presented in peanut WRKY proteins. Furthermore, most of zinc-finger motifs in N-terminal of group I are CX_4_CX_22_HX_1_H but not CX_4_CX_23_HX_1_H. In other words, N-terminal zinc-finger motif contained one more amino acid residue between the second C and the first H in the zinc-finger structure than that in C-terminus (Tables S1, S2). On the other hand, CX_5_CX_23_HX_1_H always located in fabaceous group I WRKY proteins (Song and Nan, 2014; Song et al., 2014). However, we found AdWRKY3 (group I) contained CX_7_CX_23_HX_1_C zinc-finger motif. This type of zinc-finger structure was also found in *Oryza sativa* group I WRKY (Ross et al., 2007), indicating CX_7_CX_23_HX_1_C zinc-finger structure has appeared before the divergent of gramineous and leguminous species.

AdWRKY10, AdWRKY37, and AiWRKY28, belonged to group III, contained the nucleotide-binding site-leucine-rich repeat (NBS-LRR) domain. Previous studies showed that plants contained a larger number of NBS-LRR proteins to confer resistance to diverse pathogens (Jones and Dangl, 2006; McHale et al., 2006). It is known that some WRKY proteins contain NBS-LRR domain (Deslandes et al., 2003; Shen et al., 2007; Chang et al., 2009), and group III WRKY proteins are mainly involved in plant disease resistance (Kalde et al., 2003; Eulgem and Somssich, 2007). Our results suggested AdWRKY10, AdWRKY37, and AiWRKY28 were possibly involved in disease resistance.

### Origin of WRKY protein in leguminous plants remain to be solved

**Figure 1:**
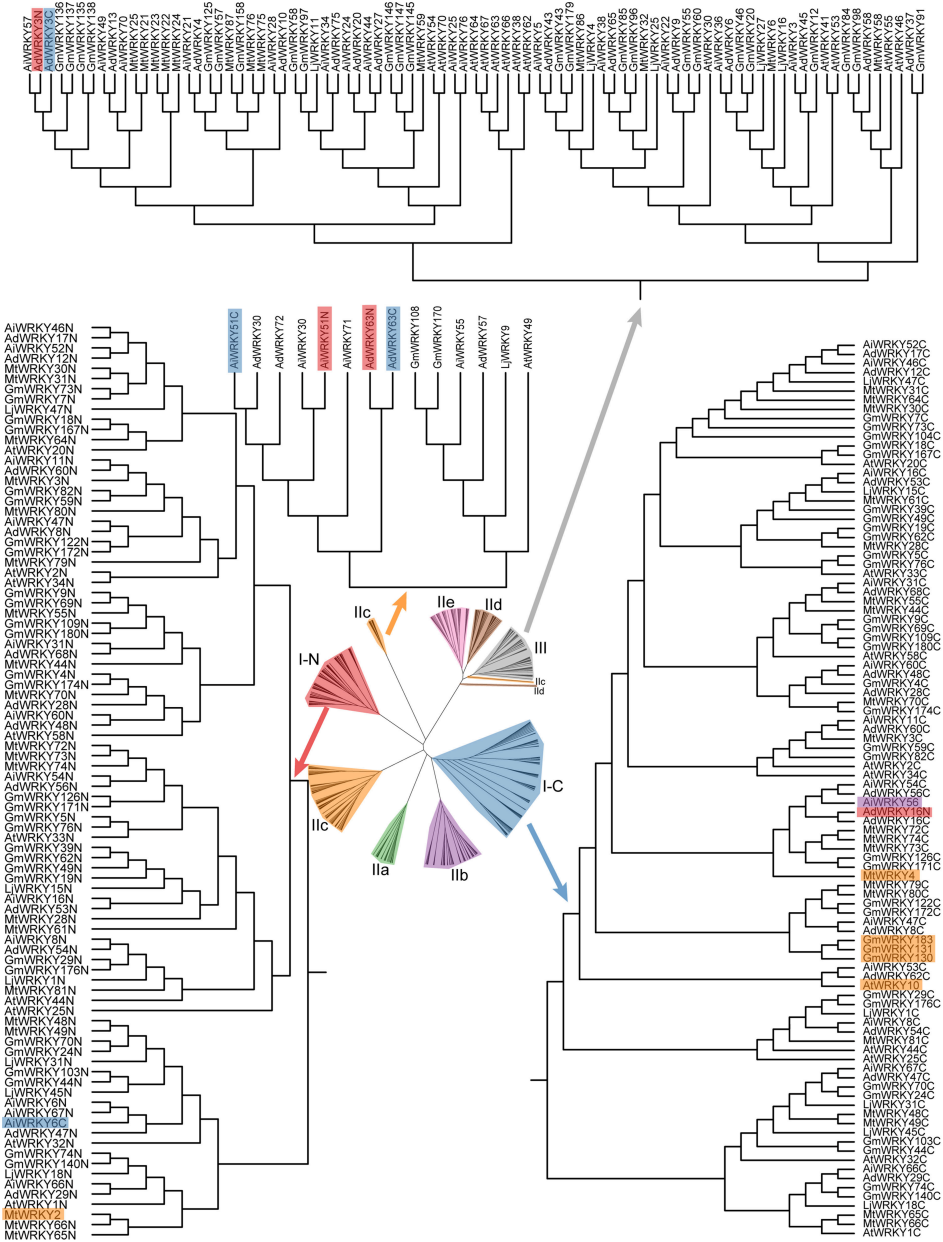
**Phylogenetic tree of WRKY domains in six plants**. The phylogenetic tree was constructed using MEGA 6.0 by the Neighbor-Joining (NJ) method with 1000 bootstrap replicates.

The phylogenetic relationship from WRKY domains of AtWRKY, MtWRKY, LjWRKY, GmWRKY, AdWRKY, and AiWRKY is consistent with the results of previous study (Zhang and Wang, 2005). The findings revealed that based on the phylogenetic trees WRKY sequences could be classified into eight subgroups: I-N, I-C, IIa, IIb, IIc, IId, IIe, and III (Zhang and Wang, 2005). However, phylogenetic relationship of MtWRKY and LjWRKY showed that some group II or III members were nested in subgroup I-N or subgroup I-C (Song and Nan, 2014; Song et al., 2014). As shown in Figure 1, subgroup I-C contained other group members, including AiWRKY56, MtWRKY4, GmWRKY130, GmWRKY131, GmWRKY183, and AtWRKY10. It indicated that the mixture was not only found in leguminous plants, but also in other dicotyledonous plants. Subgroup I-N contained MtWRKY2 from IIc, and AiWRKY6c from I-C. Group I proteins were found in other groups. For example, two WRKY domains of each AdWRKY63 and AiWRKY51 were found in subgroup IIc. Two WRKY domains of AdWRKY3 were located in subgroup III in the phylogenetic tree. These findings indicated that the origin of leguminous WRKY proteins is still to be clarified.

There are different opinions about origination of each type of WRKY proteins. In the beginning, researchers considered that the group II and III WRKY domains are the descendants originated from C-terminal WRKY domain of group I (Eulgem et al., 2000; Zhang and Wang, 2005). However, Zhu et al. (2013) found that *Triticum aestivum* IIc WRKY domains originated from the N-terminal WRKY domain of group I. Song et al. (2014) found that some *L. japonicus* and *M. truncatula* group II WRKY proteins derived from the N-terminal WRKY domain of group I. Wei et al. (2012a) demonstrated that group I WRKY protein firstly appeared in monocotyledons, followed by group III and II. In contrast, some researchers do not agree that group I WRKY protein is the ancestral member. Brand et al. (2013) reported that group I and other WRKY proteins likely originated from subgroup IIc. Recently, Rinerson et al. (2015) proposed two alternative hypotheses of WRKY protein evolution, “Group I Hypothesis” and “IIa + b Separate Hypothesis”. “Group I Hypothesis” considered all WRKY proteins evolving from C-terminal WRKY domains of group I proteins, whereas the “IIa + b Separate Hypothesis” considered groups IIa and IIb evolving directly from a single domain algal gene separated from group I-derived lineage (Rinerson et al., 2015).

### *WRKY* orthologous were located in syntenic locus of two *Arachis* genomes

**Figure 2:**
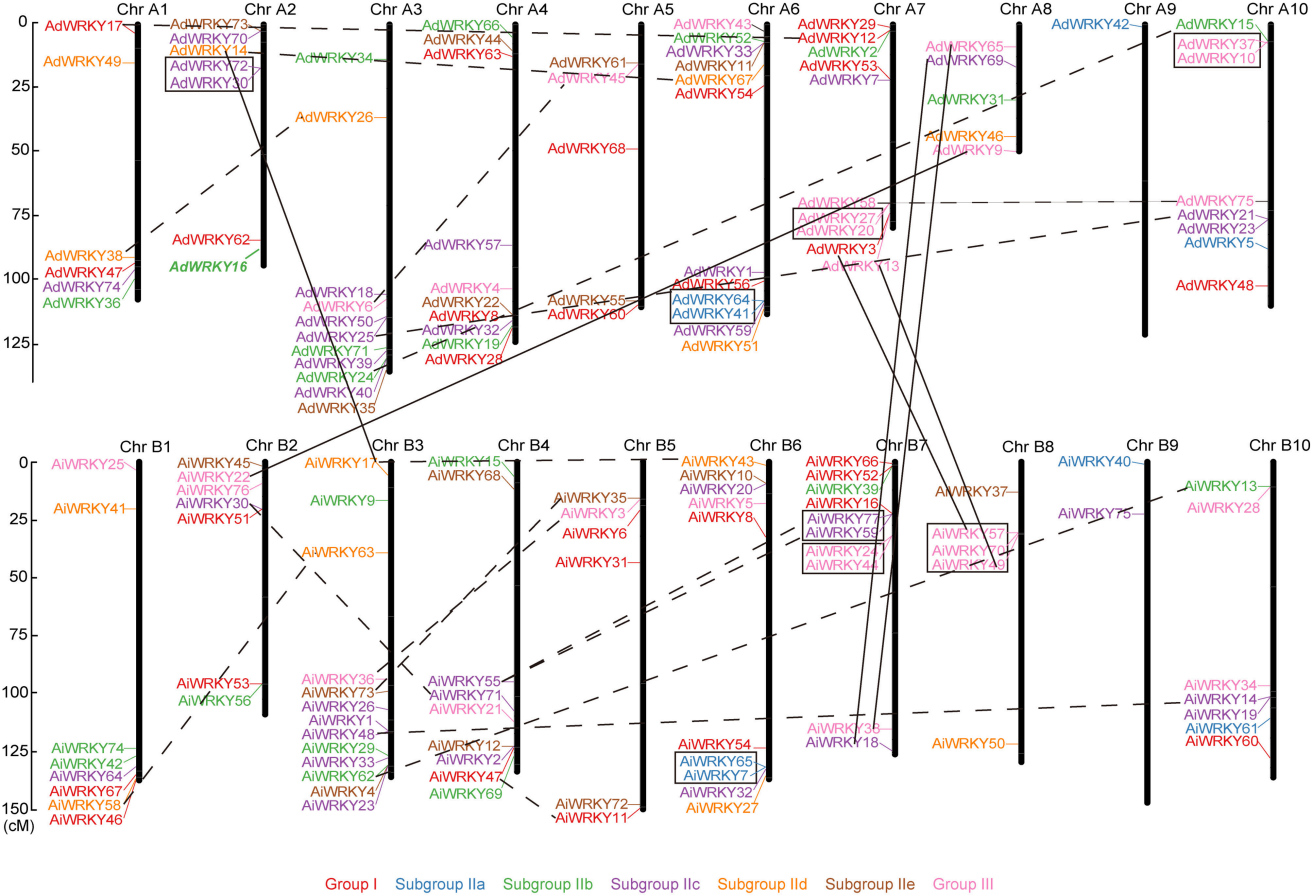
**Chromosomal location of *AdWRKY* and *AiWRKY* genes**. The chromosome numbers were shown at the top of each chromosome (black bars). The location of each *WRKY* gene was pointed out by a line. The solid lines indicated orthologous genes locations on the two peanut species' chromosomes. The dotted lines indicated the segmental duplicated genes. The black boxes indicated the tandem duplication genes. Italic indicated predicated chromosomal location in this study.

As shown in Figure 2, *AdWRKY* and *AiWRKY* genes were randomly distributed across 10 chromosomes. Chromosome A6 contained the largest number of *WRKY* genes (12), while chromosome A9 contained the least number of WRKY genes (1) in *A. duranensis*. In *A. ipaënsis*, 13 genes were distributed in chromosome B3, whereas only two *WRKY* genes were found in chromosome B9. The length of *A. duranensis* chromosome A7 (79.13 cM) and A8 (49.46 cM) is shorter than that of *A. ipaënsis* chromosome B7 (126.23 cM) and B8 (129.61 cM). However, chromosome 7 and 8 in these two species all contained 10 and 5 *WRKY* genes, respectively (Figure 2). There was no positive correlation between the chromosome length and the number of *WRKY* genes. In soybean, we found that most *GmWRKY* genes were located in the chromosome arm (Song et al., 2016). Same distribution pattern was observed in peanut. Previous study found more colinearity between *Arachis* and *Glycine* to compare with other leguminous plants (Nagy et al., 2012). The ends of chromosome exhibited stronger synteny than the central regions of chromosomes (Nagy et al., 2012).

In this study, we detected 63 orthologous gene pairs according to the phylogenetic relationship of *AdWRKY* and *AiWRKY* genes (Table 2, Figure S3). Among which 56 orthologous gene pairs were found on the syntenic locus in *A. duranensis* and *A. ipaënsis* chromosome (Table 2, Tables S1, S2). However, the location of six *AdWRKY* genes (*AdWRKY3, 9, 13, 14, 65*, and *69*) did not correspond to their orthologous gene in *A. ipaënsis* (*AiWRKY57, 22, 49, 17, 38*, and *18*; Figure 2, Table 2). Physical mapping revealed that *WRKY* genes in *Gossypium arboreum* were not located in the corresponding chromosomes of *G. raimondii*, suggesting the occurrence of large chromosome rearrangement in the diploid cotton genomes (Ding et al., 2015).

**Table 2:** **The information of orthologous genes in *AdWRKY* and *AiWRKY***.

**Gene paris**	**Groups**	**Chromosome**	**CDS identity (%)**	**Protein identity (%)**
AdWRKY2-AiWRKY39	IIb-IIb	7−7	96.64	91.52
AdWRKY3-AiWRKY57	I-III	7−8	95.37	94.34
AdWRKY4-AiWRKY21	III-III	4−4	97.92	95.69
AdWRKY5-AiWRKY61	IIa-IIa	10−10	98.23	97.17
AdWRKY6-AiWRKY36	III-III	3−3	98.10	97.55
AdWRKY7-AiWRKY59	IIc-IIc	7−7	98.57	97.23
AdWRKY8-AiWRKY47	I-I	4−4	98.82	95.86
AdWRKY9-AiWRKY22	III-III	8−2	98.73	98.08
AdWRKY10-AiWRKY28	III-III	10−10	98.61	97.22
AdWRKY11-AiWRKY10	IIe-IIe	6−6	98.68	98.57
AdWRKY12-AiWRKY52	I-I	7−7	96.83	95.94
AdWRKY13-AiWRKY49	III-III	7−8	99.29	73.31
AdWRKY14-AiWRKY17	IId-IId	2−3	99.15	99.15
AdWRKY15-AiWRKY13	IIb-IIb	10−10	95.74	93.47
AdWRKY16-AiWRKY56	I-IIb	*2*−2	98.04	85.65
AdWRKY17-AiWRKY46	I-I	1−1	99.23	99.18
AdWRKY18-AiWRKY26	IIc-IIc	3−3	98.94	98.72
AdWRKY19-AiWRKY69	IIb-IIb	4−4	97.18	96.82
AdWRKY20-AiWRKY24	III-III	7−7	95.50	88.99
AdWRKY21-AiWRKY14	IIc-IIc	10−10	97.94	94.79
AdWRKY22-AiWRKY12	IIe-IIe	4−4	97.19	97.09
AdWRKY23-AiWRKY19	IIc-IIc	10−10	98.63	92.79
AdWRKY24-AiWRKY62	IIb-IIb	3−3	94.53	90.36
AdWRKY25-AiWRKY48	IIc-IIc	3−3	96.24	94.59
AdWRKY26-AiWRKY63	IId-IId	3−3	98.66	99.07
AdWRKY27-AiWRKY44	III-III	7−7	97.96	95.80
AdWRKY29-AiWRKY66	I-I	7−7	98.94	98.41
AdWRKY33-AiWRKY20	IIc-IIc	6−6	98.30	81.29
AdWRKY34-AiWRKY9	IIb-IIb	3−3	96.52	90.89
AdWRKY36-AiWRKY42	IIb-IIb	1−1	98.59	96.70
AdWRKY38-AiWRKY58	IId-IId	1−1	96.42	83.38
AdWRKY39-AiWRKY33	IIc-IIc	3−3	99.42	98.82
AdWRKY40-AiWRKY23	IIc-IIc	3−3	96.63	96.99
AdWRKY41-AiWRKY7	IIa-IIa	6−6	99.13	98.87
AdWRKY42-AiWRKY40	IIa-IIa	9−9	99.07	99.38
AdWRKY43-AiWRKY5	III-III	6−6	98.62	97.24
AdWRKY44-AiWRKY68	IIe-IIe	4−4	97.71	97.57
AdWRKY45-AiWRKY3	III-III	5−5	98.81	96.41
AdWRKY46-AiWRKY50	IId-IId	8−8	98.94	98.26
AdWRKY47-AiWRKY67	I-I	1−1	98.19	96.89
AdWRKY48-AiWRKY60	I-I	10−10	96.47	92.45
AdWRKY49-AiWRKY41	IId-IId	1−1	97.44	97.80
AdWRKY50-AiWRKY1	IIc-IIc	3−3	98.84	98.83
AdWRKY51-AiWRKY27	IId-IId	6−6	97.46	95.36
AdWRKY53-AiWRKY16	I-I	7−7	98.00	97.57
AdWRKY54-AiWRKY8	I-I	6−6	98.50	98.07
AdWRKY55-AiWRKY72	IIe-IIe	5−5	97.19	96.34
AdWRKY56-AiWRKY54	I-I	6−6	98.28	98.34
AdWRKY57-AiWRKY55	IIc-IIc	4−4	95.60	93.26
AdWRKY59-AiWRKY32	IIc-IIc	6−6	97.87	95.91
AdWRKY60-AiWRKY11	I-I	5−5	98.42	98.28
AdWRKY61-AiWRKY35	IIe-IIe	5−5	97.06	96.88
AdWRKY62-AiWRKY53	I-I	2−2	97.31	93.74
AdWRKY64-AiWRKY65	IIa-IIa	6−6	96.84	94.90
AdWRKY65-AiWRKY38	III-III	8−7	98.17	97.69
AdWRKY66-AiWRKY15	IIb-IIb	4−4	96.55	96.61
AdWRKY67-AiWRKY43	IId-IId	6−6	99.35	99.34
AdWRKY68-AiWRKY31	I-I	5−5	98.80	93.91
AdWRKY69-AiWRKY18	IIc-IIc	8−7	96.02	68.82
AdWRKY71-AiWRKY29	IIb-IIb	3−3	93.91	92.68
AdWRKY73-AiWRKY45	IIe-IIe	2−2	97.70	95.97
AdWRKY74-AiWRKY64	IIc-IIc	1−1	96.39	95.54
AdWRKY75-AiWRKY34	III-III	10−10	98.68	87.46

*Italic indicated predicated chromosome*.

*AdWRKY16* gene has temporarily no precise location information in chromosome. Its orthologous gene, *AiWRK56*, was located in lower end of chromosome B2 (Figure 2). We speculated that *AdWRKY16* gene might be distributed in the corresponding locus on chromosome A2 (Figure 2, italics bold).

### Segmental duplication events played a major role in *Arachis WRKY* gene evolution

Gene duplication events occurred universally in *WRKY* genes (Cannon et al., 2004). Duplicated genes were considered to be the raw materials for the evolution of new biological functions and played crucial roles in adaption (Nei and Rooney, 2005). In this study, we employed duplicated *GmWRKY* genes (Yin et al., 2013) as query sequences to construct the molecular phylogenetic tree with *AdWRKY* and *AiWRKY* genes, respectively (Figures S4, S5). Results showed that gene duplication was detected for 22 *AdWRKY* and 26 *AiWRKY* genes (Table 3). Eight and 14 *AdWRKY* genes were experienced four tandem duplication and seven segmental duplication events, respectively (Figure 2, Table 3). Among the *AiWRKY* genes, four tandem duplication events with nine genes and 10 segmental duplication events with 17 genes were observed (Figure 2, Table 3). These results indicated that segmental duplication events played a major driving force for *AdWRKY* and *AiWRKY* evolution. This result is consistent with that most *WRKY* genes in soybean were derived from segmental duplication (Yin et al., 2013). However, the birth of *WRKY* gene in cotton and cocao genomes were considered through segmental duplication followed by tandem duplication (Dou et al., 2014; Ding et al., 2015). We calculated the Ks and Ka values and found that all duplicated *AdWRKY* and *AiWRKY* gene pairs with a ω value of < 1. This result indicated that purifying selection occurred on these duplicated gene pairs, which was agreed to the results of *Brassica rapa* (Tang et al., 2014).

**Table 3:** **Ka/Ks calculation of each duplicated *AdWRKY* and *AiWRKY* genes pairs**.

**Duplication gene pairs**	**S**	**N**	**Ka**	**Ks**	**Ka/Ks**	**Duplicated type**	**Selection pressure**
***ARACHIS DURANENSIS***							
AdWRKY58-AdWRKY75	187.0	674.0	0.9273	4.4201	0.2098	Segmental	Purify selection
AdWRKY64-AdWRKY41	138.4	587.6	0.6971	3.6742	0.1897	Tandem	Purify selection
AdWRKY6-AdWRKY45	258.5	740.5	0.3031	0.6350	0.4773	Segmental	Purify selection
AdWRKY14-AdWRKY67	102.6	353.4	0.0343	0.6434	0.0533	Segmental	Purify selection
AdWRKY12-AdWRKY17	410.9	1245.1	0.1650	0.7778	0.2121	Segmental	Purify selection
AdWRKY26-AdWRKY38	218.0	634.0	0.1855	1.3704	0.1353	Segmental	Purify selection
AdWRKY30-AdWRKY72	153.5	416.5	0.0350	0.0719	0.4873	Tandem	Purify selection
AdWRKY10-AdWRKY37	694.6	2098.4	0.1355	0.2489	0.5445	Tandem	Purify selection
AdWRKY15-AdWRKY24	214.7	736.3	0.3077	1.2914	0.2382	Segmental	Purify selection
AdWRKY20-AdWRKY27	158.6	570.4	0.3698	0.6555	0.5641	Tandem	Purify selection
AdWRKY21-AdWRKY25	31.9	160.1	0.1534	0.9160	0.1674	Segmental	Purify selection
***ARACHIS IPAËNSIS***							
AiWRKY7-AiWRKY65	142.1	589.9	0.6849	3.7187	0.1842	Tandem	Purify selection
AiWRKY3-AiWRKY36	265.7	733.3	0.3034	0.6263	0.4845	Segmental	Purify selection
AiWRKY17-AiWRKY43	209.8	819.2	0.1174	0.8335	0.1408	Segmental	Purify selection
AiWRKY58-AiWRKY63	221.8	612.2	0.1670	1.3355	0.1250	Segmental	Purify selection
AiWRKY30-AiWRKY71	105.9	323.1	0.2019	0.4765	0.4238	Segmental	Purify selection
AiWRKY11-AiWRKY47	428.4	1428.6	0.5001	54.6848	0.0091	Segmental	Purify selection
AiWRKY13-AiWRKY62	255.2	833.8	0.3370	1.2712	0.2651	Segmental	Purify selection
AiWRKY35-AiWRKY73	70.7	235.3	0.3145	1.7563	0.1791	Segmental	Purify selection
AiWRKY59-AiWRKY77	107.0	433.0	0.2700	0.7250	0.3724	Tandem	Purify selection
AiWRKY59-AiWRKY55	161.4	582.6	0.8853	2.9049	0.3048	Segmental	Purify selection
AiWRKY77-AiWRKY55	173.2	591.8	0.7702	3.4467	0.2235	Segmental	Purify selection
AiWRKY24-AiWRKY44	133.6	466.4	0.2987	0.7527	0.3968	Tandem	Purify selection
AiWRKY49-AiWRKY57	97.3	376.7	0.4404	1.2697	0.3468	Tandem	Purify selection
AiWRKY49-AiWRKY70	132.4	443.6	0.3201	0.5728	0.5588	Tandem	Purify selection
AiWRKY57-AiWRKY70	59.0	238.0	0.3353	1.3690	0.2450	Tandem	Purify selection
AiWRKY14-AiWRKY48	32.0	160.0	0.1449	0.9221	0.1572	Segmental	Purify selection

*S, number of synonymous sites; N, number of non-synonymous sites; Ka, non-synonymous substitution rate; Ks, synonymous substitution*.

### Different selection pressure in two wild peanut species

Site model and branch-site model were used to estimate the selection pressure of AdWRKY and AiWRKY proteins. The results of site model showed that AdWRKY and AiWRKY proteins underwent purifying pressure during evolution (Table S3). It was showed that cotton WRKY proteins within each group are under strong purifying pressure (Ding et al., 2015). Li et al. (2015) found a strong acting of purifying selection in the evolution of *Salvia miltiorrhiza* WRKY proteins. Similarly, Group III WRKY proteins from *L. japonicus* (Song et al., 2014), *M. truncatula* (Song and Nan, 2014) and *C. sativus* (Ling et al., 2011) were considered to be under purifying selection. Although, AdWRKY and AiWRKY proteins underwent purifying selection, positive selected sites in group I (1 site), subgroup IIc (4 sites), IIe (5 sites), and group III (7 sites) AdWRKY proteins were detected using branch-site model (Table S4). In AiWRKY proteins, positive selected sites were discovered in group I (1 site), subgroup IIe (3 sites), and group III (1 site) (Table S5). More positive selected sites were detected in AdWRKY proteins than in AiWRKY proteins, indicating the existence of different degrees of positive selective pressure in AdWRKY and AiWRKY proteins. Purifying selection may generate genes with conserved functions or pseudogenization, but hard for neofunctionalization or subfunctionalization (Zhang, 2003). Based on this consideration, we tentatively suggested that AdWRKY group III and AiWRKY sungroup IIe genes may have various biological functions, instead, AdWRKY subgroup IIa, IIb and IId and AiWRKY subgroup IIa, IIb, IIc, and IId genes possibly have conserved biological functions.

Positively-selected sites were found using branch-site model. In cotton, group IIa and IId WRKY proteins were found many positive selection sites, while group I and subgroup IIc proteins had the lowest and no positive selection sites was found in cotton WRKY group III (Ding et al., 2015). GmWRKY group I, IIc, IIe, and III WRKY proteins had positive selection sites (Yin et al., 2013). The group IIc and III WRKY proteins from eggplant (*Solanum melongena*) and turkey berry (*Solanum torvum*) detected positive selection (Yang et al., 2015). Li et al. (2015) found that positive selection sites were presented in SmWRKY subgroup IIb, IIc, IId, IIe, and group III genes, while no positive selection site was detected in group I and subgroup IIa.

### *Arachis WRKY* gene activities respond to SA and MeJA

We intended to translate the knowledge from above wild ancestral peanut study to benefit disease resistance research in economic-important cultivated peanut. To obtain specific AdWRKY and AiWRKY that potentially involved in both SA and JA signaling pathways, we constructed phylogenetic tree using AdWRKY, AiWRKY, and AtWRKY proteins, respectively (Figures S6, S7), and then deduced SA- and JA-relative AdWRKY and AiWRKY proteins were determined if proteins were classified in the same clade of specific AtWRKY proteins known for being involved in both SA and JA signaling pathways (Dong et al., 2003; Schluttenhofer et al., 2014). The deduced SA- and JA-related *AdWRKY* and *AiWRKY* genes were used as query sequences to identify cultivated peanut *WRKY* genes (*AhWRKY*) using local BLASTN against the peanut transcriptome database and the peanut genome (unpublished data). Ultimately, we found 13 *AhWRKY* genes, named *AhWRKY1* to *AhWRKY13* (Tables S6, S7), were deduced to be potentially involved in both SA and JA signaling pathways in cultivated peanut.

**Figure 3:**
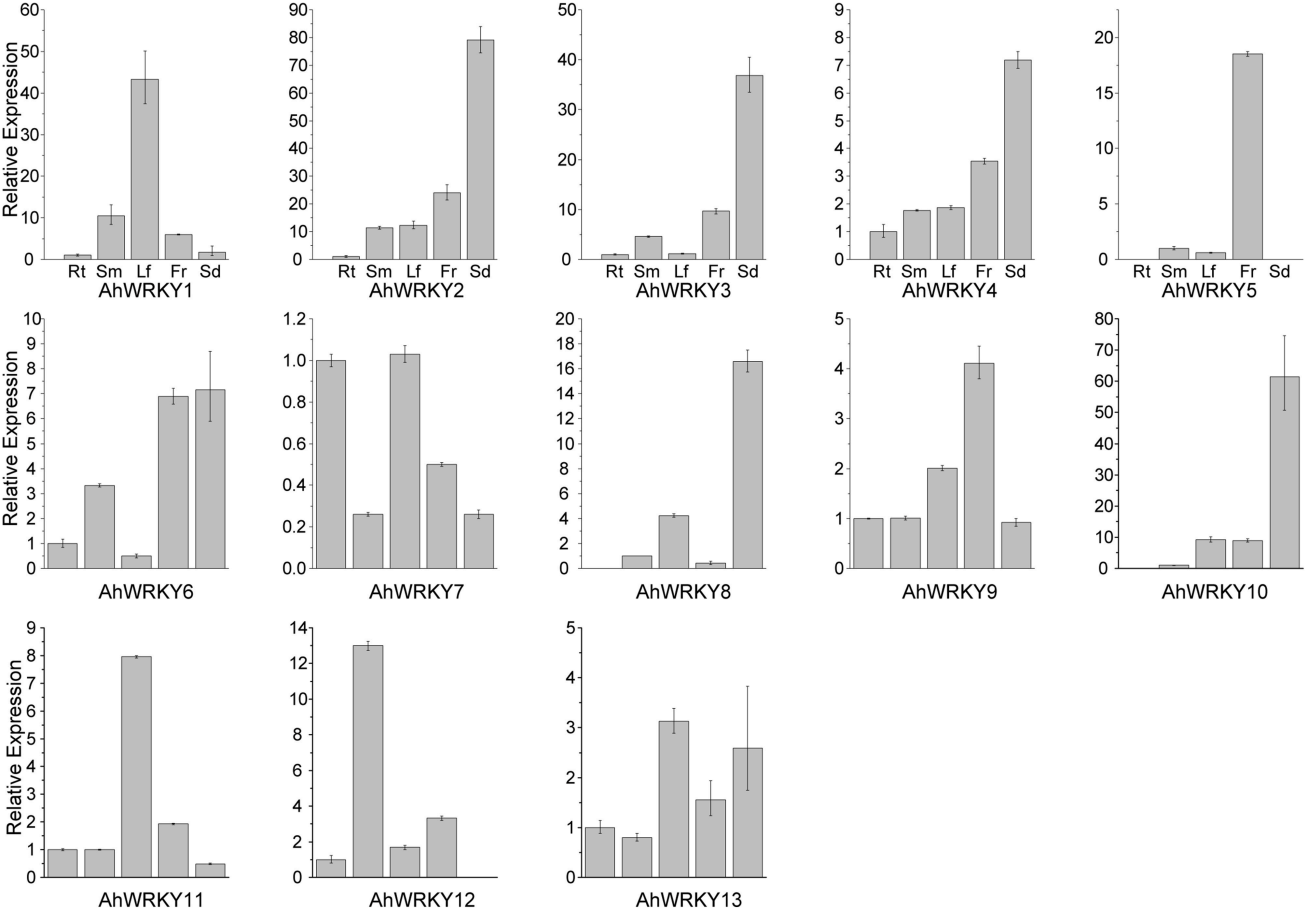
**The expression pattern of *AhWRKY* genes in different tissues**. The abbreviation Rt, Sm, Lf, Fr, and Sd in the tissue label indicated root, stem, leaf, flower and seed, respectively.

We examined the transcriptional levels of these 13 genes in five different tissues by qRT-PCR and found each gene could be detected in at least one of the five tested tissues (Figure 3). Previous studies demonstrated that most *WRKY* genes expressed constitutively (Huang et al., 2012; Wei et al., 2012b; Dou et al., 2014; Jiang et al., 2014). The expression of *AhWRKY1, 2, 3, 4, 5, 6, 7, 9, 11*, and *13* genes could be detected in all five tissues (Figure 3). The expression of *AhWRKY5, 8* and *10* genes were not detected in root; the expression of *AhWRKY5* and *12* genes was not detected in seed. All 13 *AhWRKY* genes were expressed in stem, leaf and flower (Figure 3). *AhWRKY2, 3, 4, 6, 8, 10*, and *13* genes were mainly expressed in seed, indicating their function in seed development. Ding et al. (2015) showed that most cotton *WRKY* genes were highly expressed in all developmental stages of seed. These genes could be good candidates for *Aspergillus flavus* resistance, because Fountain et al. (2015) found that *WRKY* genes may play important roles in maize kernels against *A. flavus* infection.

**Figure 4:**
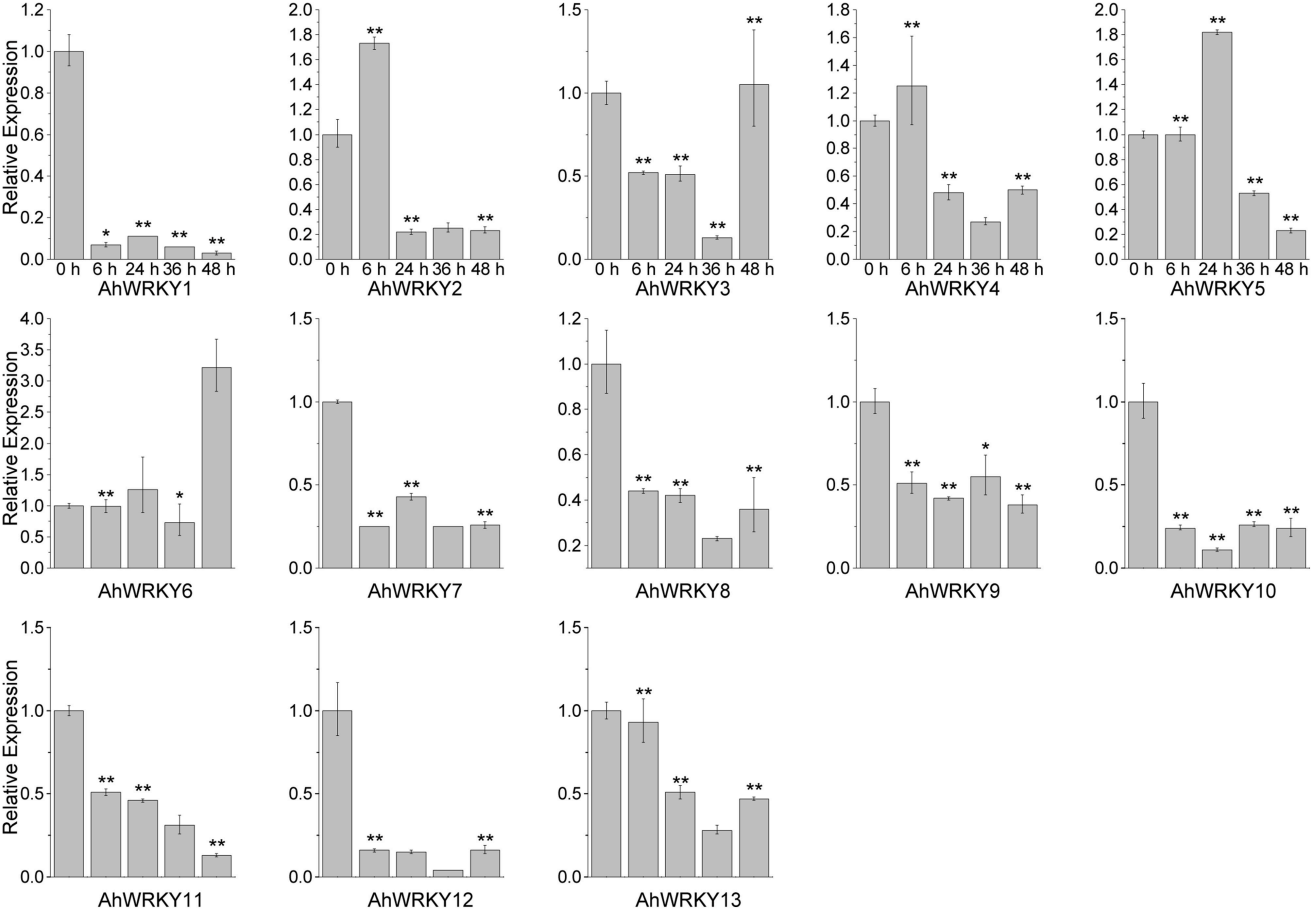
**The expression pattern of *AhWRKY* genes after SA treatment**. The Y-axis indicates the relative expression level; X-axis (0, 6, 24, 36, and 48 h) indicated hours of SA treatment. The standard errors are plotted using vertical lines. ^*^ and ^**^ mean significant difference at *P* < 0.05 and *P* < 0.01, respectively.

**Figure 5:**
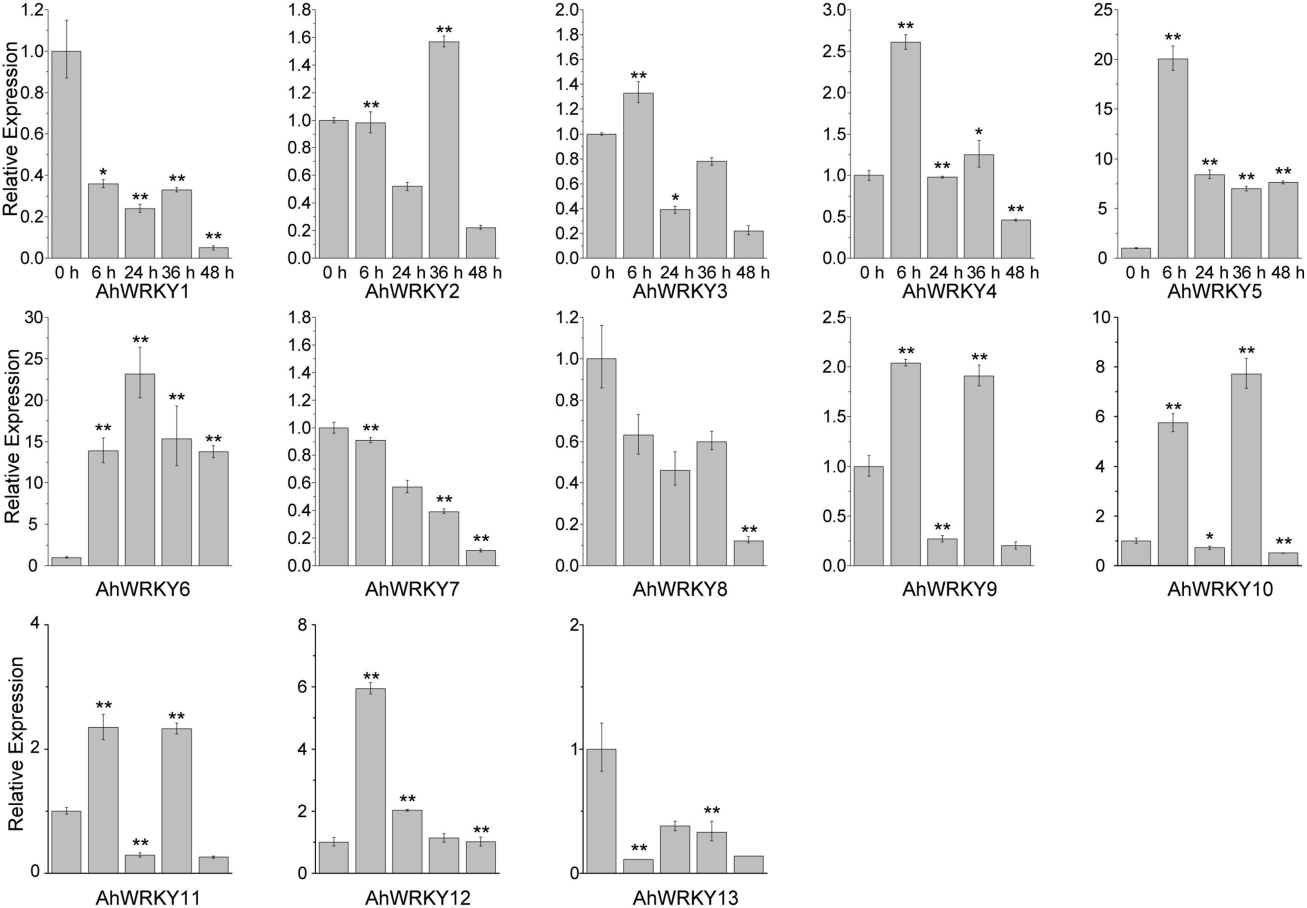
**The expression pattern of *AhWRKY* genes after MeJA treatment**. The Y-axis indicates the relative expression level; X-axis (0, 6, 24, 36, and 48 h) indicated hours of MeJA treatment. The standard errors are plotted using vertical lines. ^*^ and ^**^ mean significant difference at *P* < 0.05 and *P* < 0.01, respectively.

Then we asked if gene activities of 13 *AhWRKY* genes were affected by SA and MeJA treatments. As shown in Figure 4, *AhWRKY1, 7, 8, 9, 10, 11, 12*, and *13* genes were down-regulated at all five time points under SA treatment. However, *AhWRKY5, 6*, and *12* genes was up-regulated and the *AhWRKY1, 7, 8*, and *13* genes were down-regulated at all five time points under MeJA treatment (Figure 5). *AhWRKY12* genes showed an opposite response to SA and MeJA treatment, while *AhWRKY1, 7, 8*, and 13 genes responded similar to these two hormones. Previous studies showed shat SA and MeJA signaling pathways often play an opposite role in defense, but synergistic effect between these phytohormones had also been reported (Mur et al., 2006). Lai et al. (2008) found that *AtWRKY4* played positive role in plant resistance to necrotrophic pathogens, while overexpression of *AtWRKY4* increased the plant susceptibility to biotrophic pathogen. However, *AtWRKY70* (Li et al., 2004) and *CaWRKY27* (Dang et al., 2014) regulated simultaneously SA and MeJA signaling pathways and acted as a positive regulator in response to pathogen.

*AhWRKY1, 7, 8, 12*, and *13* genes are orthologous genes of *AtWRKY53, 40, 53, 21*, and *3*, respectively (Table S6). *AtWRKY53* showed a strong increase in expression within the first 2 h but the transcript level greatly reduced at later time under SA treatment (Yu et al., 2001; Kalde et al., 2003). Miao and Zentgraf (2007) found that 4 h MeJA treatment reduced expression of *AtWRKY53*. *AtWRKY40* gene was not considered as a positive regulator for systemic acquired resistance (Wang et al., 2006). Knocking out the *AtWRKY40* gene led to the increased resistant to *Golovinomyces orontii* (Shen et al., 2007) and *Pseudomonas syringe* (Xu et al., 2006). Kalde et al. (2003) found that the expression of *AtWRKY21* was induced by SA. *CrWRKY36*, orthologous gene of *AtWRKY21*, was up-regulated after 2 h MeJA treatment (Schluttenhofer et al., 2014). *AtWRKY3* gene was involved in SA and JA signaling pathways. *AtWRKY3* gene played a negative role in SA signaling pathway and mediated resistance to biotrophic pathogens, while it played a positive role in MeJA mediated resistance to necrotrophic pathogen (Lai et al., 2008). Interestingly, the expression of *AhWRKY3* and *12* (paralogous genes), orthologous genes of *AtWRKY21*, responded oppositely to SA and MeJA treatments. Orthologous genes shared a conserved ancestral gene function in different species, while function of paralogous genes diversified through gene duplication (Koonin, 2005; Gabaldón and Koonin, 2013).

## Conclusions

Similar number of WRKY proteins was identified in *A. duranensis* and *A. ipaënsis*. Orthologous gene pairs were found on the identical or similar locus on chromosomes in these two species. Our results showed that segmental duplication events played a major role in *AdWRKY* and *AiWRKY* evolution. Peanut WRKY proteins were under strong purifying pressure. Similar or opposite response of peanut *WRKY* genes to SA and JA treatments was observed. Our results could help to select appropriate candidate genes for further characterization of their pathogen resistant functions in *Arachis* species.

## Author contributions

HS wrote the manuscript and performed the laboratory assays. PW performed the phylogenetic analysis and reviewed the manuscript. JL provided the value comments and revised the grammar of the manuscript. CZ provided help in analysis of qRT-PCR. YB helped to revise the manuscript. XW served as the principal investigator, facilitated the project, and assisted in manuscript preparation and revision.

### Conflict of interest statement

The authors declare that the research was conducted in the absence of any commercial or financial relationships that could be construed as a potential conflict of interest.

## Supplementary material

Figure S1 **Phylogenetic tree of AtWRKY and AdWRKY domains**. The phylogenetic tree was constructed using MEGA 6.0 by the Neighbor-Joining (NJ) method with 1000 bootstrap replicates.

Figure S2 **Phylogenetic tree of AtWRKY and AiWRKY domains**. The phylogenetic tree was constructed using MEGA 6.0 by the Neighbor-Joining (NJ) method with 1000 bootstrap replicates.

Figure S3 **Phylogenetic tree of AdWRKY and AiWRKY proteins**. The phylogenetic tree was constructed using MEGA 6.0 by the Neighbor-Joining (NJ) method with 1000 bootstrap replicates.

Figure S4 **Phylogenetic tree of AdWRKY and GmWRKY proteins**. The phylogenetic tree was constructed using MEGA 6.0 by the Neighbor-Joining (NJ) method with 1000 bootstrap replicates.

Figure S5 **Phylogenetic tree of AiWRKY and GmWRKY proteins**. The phylogenetic tree was constructed using MEGA 6.0 by the Neighbor-Joining (NJ) method with 1000 bootstrap replicates.

Figure S6 **Phylogenetic tree of *AtWRKY* and *AiWRKY* genes involved in both response to SA and MeJA**. The phylogenetic tree was constructed using MEGA 6.0 by the Neighbor-Joining (NJ) method with 1000 bootstrap replicates.

Figure S7 **Phylogenetic tree of *AtWRKY* and *AiWRKY* genes involved in both response to SA and MeJA**. The phylogenetic tree was constructed using MEGA 6.0 by the Neighbor-Joining (NJ) method with 1000 bootstrap replicates.

Table S1 ***Arachis duranensis WRKY* genes identified in the V14167.a1.M1**.

Table S2 ***Arachis ipaënsis WRKY* genes identified in the K30076.a1.M1**.

Table S3 **Analysis of positive selection among different codons of AdWRKY and AiWRKY using site model**. ω, Ka/Ks value; ω0, Ka/Ks value in 0 category; ω1, Ka/Ks value in the first category; ω2, Ka/Ks value in the second category; p, the number of free parameters for the ω ratios; p0, the number of free parameters for the ω0 ratios; p1, the number of free parameters for the ω1 ratios; p2, the number of free parameters for the ω2 ratios; q, the parameters of the beta distribution.

Table S4 **Tests for selection among codons of AdWRKY using branch-site models**. ω, Ka/Ks value; ωb0, Ka/Ks value in background under model 0; ωb1, Ka/Ks value in background under model 1; ωb2a, Ka/Ks value in background under model 2a; ωb2b, Ka/Ks value in background under model 2b; ωf0, Ka/Ks value in foreground under model 0; ωf1, Ka/Ks value in foreground under model 1; ωf2a, Ka/Ks value in foreground under model 2a; ωf2b, Ka/Ks value in foreground under model 2b; p, the number of free parameters for the ω ratios; p0, the number of free parameters for the ω ratios under model 0; p1, the number of free parameters for the ω ratios under model 1; p2a, the number of free parameters for the ω ratios under model 2a; p2b, the number of free parameters for the ω ratios under model 2b.

Table S5 **Tests for selection among codons of AiWRKY using branch-site models**. ω, Ka/Ks value; ωb0, Ka/Ks value in background under model 0; ωb1, Ka/Ks value in background under model 1; ωb2a, Ka/Ks value in background under model 2a; ωb2b, Ka/Ks value in background under model 2b; ωf0, Ka/Ks value in foreground under model 0; ωf1, Ka/Ks value in foreground under model 1; ωf2a, Ka/Ks value in foreground under model 2a; ωf2b, Ka/Ks value in foreground under model 2b; p, the number of free parameters for the ω ratios; p0, the number of free parameters for the ω ratios under model 0; p1, the number of free parameters for the ω ratios under model 1; p2a, the number of free parameters for the ω ratios under model 2a; p2b, the number of free parameters for the ω ratios under model 2b.

Table S6 **The information of *AhWRKY* genes**.

Table S7 **qRT-PCR primers in this study**.
