# Supplementary figures and images for: Genome-Wide Identification and Characterization of WRKY Gene Family in Peanut
Source: Front Plant Sci. 2016 Apr 26;7:534. doi: 10.3389/fpls.2016.00534 (PMC4845656; doi:10.3389/fpls.2016.00534)

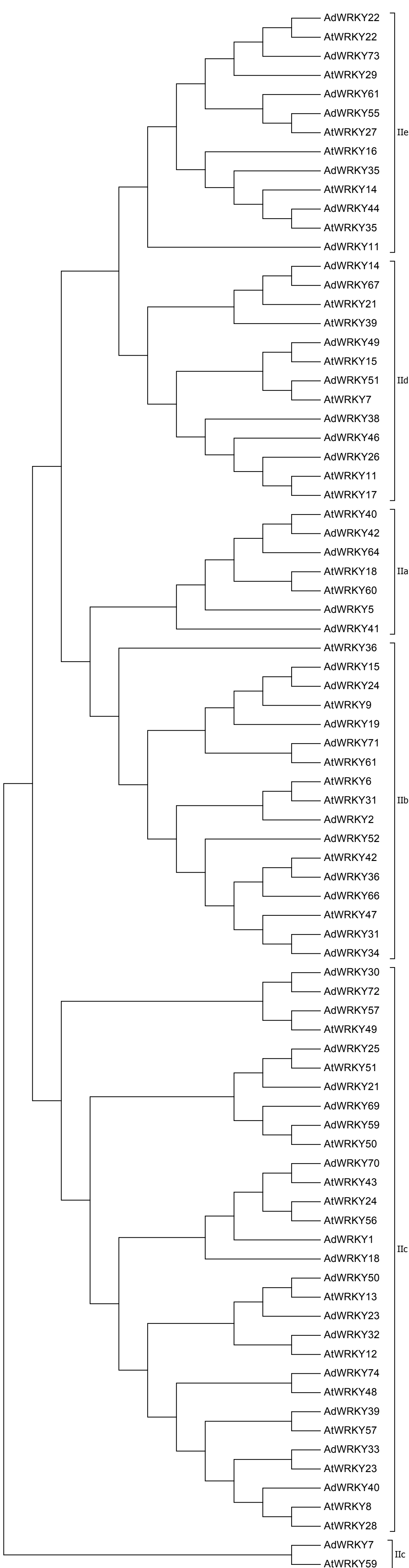

Supplement: Figure S1 — Phylogenetic tree of AtWRKY and AdWRKY domains. The phylogenetic tree was constructed using MEGA 6.0 by the Neighbor-Joining (NJ) method with 1000 bootstrap replicates. [file Image1.PDF]

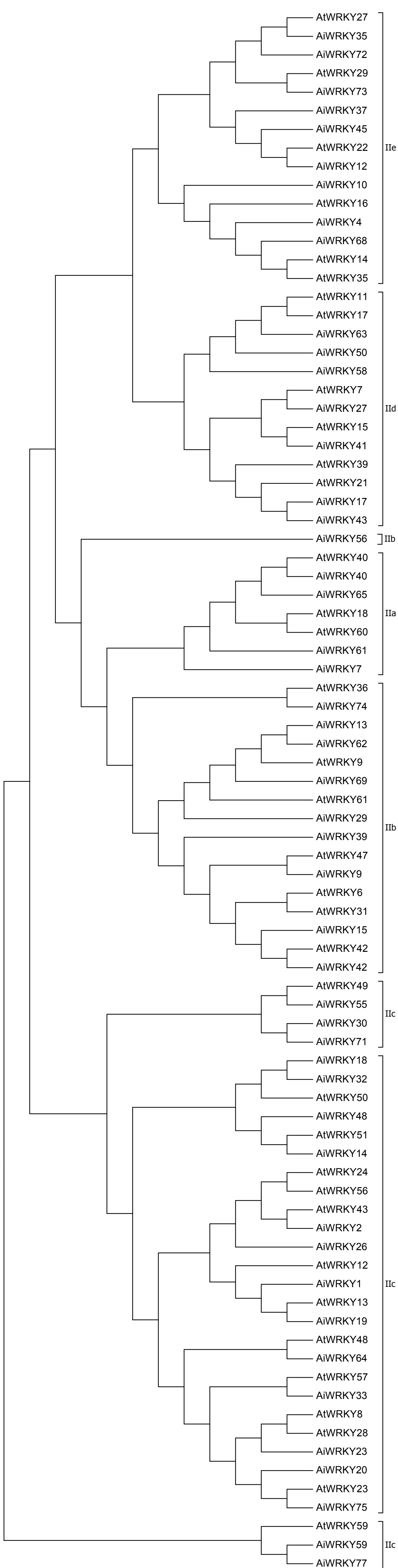

Supplement: Figure S2 — Phylogenetic tree of AtWRKY and AiWRKY domains. The phylogenetic tree was constructed using MEGA 6.0 by the Neighbor-Joining (NJ) method with 1000 bootstrap replicates. [file Image2.PDF]

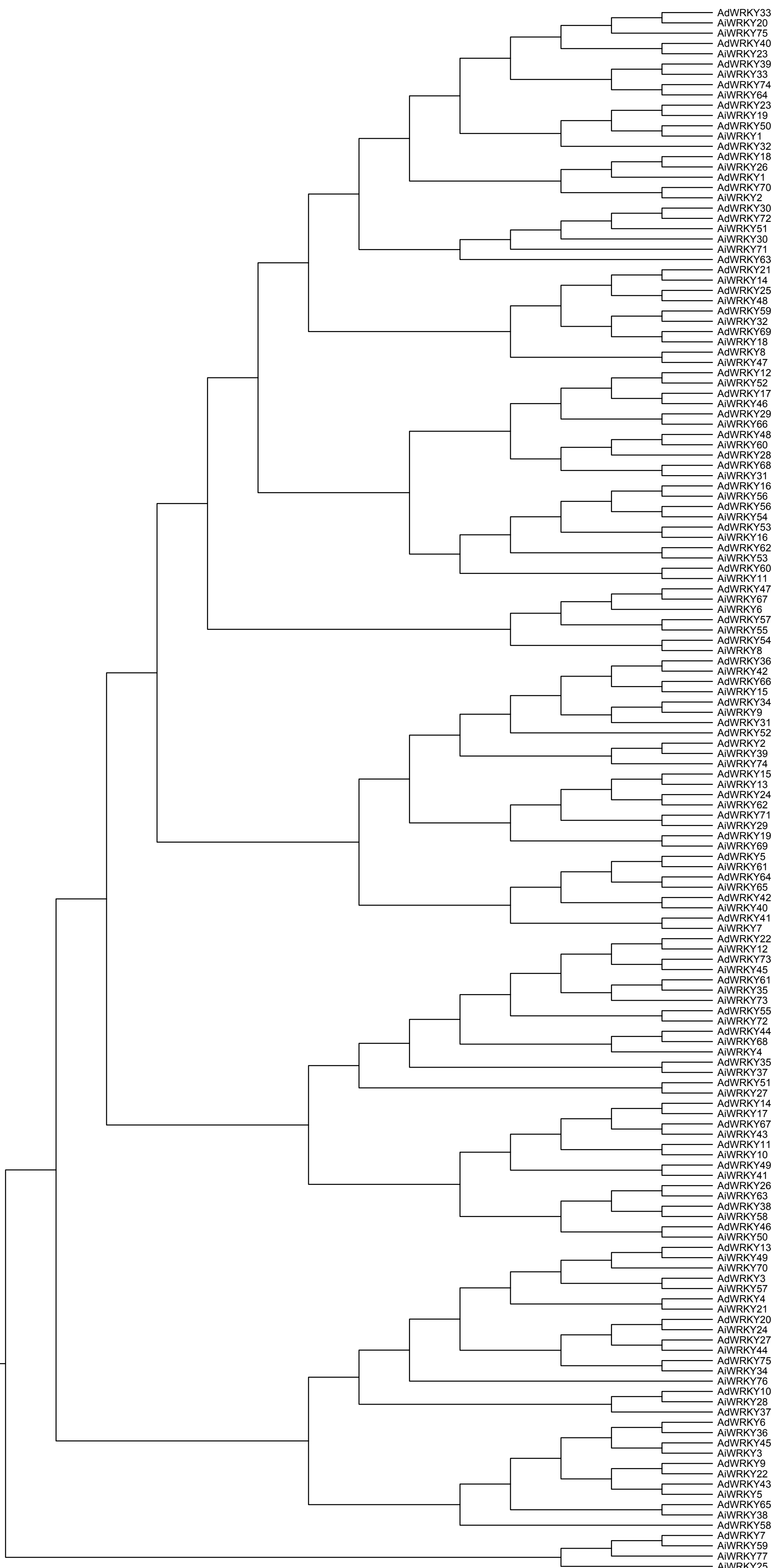

Supplement: Figure S3 — Phylogenetic tree of AdWRKY and AiWRKY proteins. The phylogenetic tree was constructed using MEGA 6.0 by the Neighbor-Joining (NJ) method with 1000 bootstrap replicates. [file Image3.PDF]

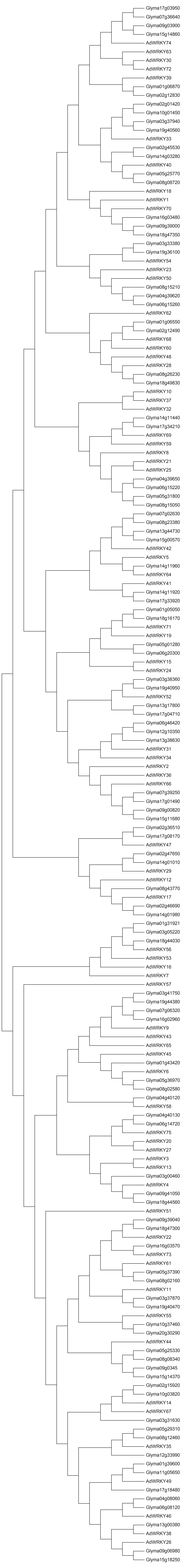

Supplement: Figure S4 — Phylogenetic tree of AdWRKY and GmWRKY proteins. The phylogenetic tree was constructed using MEGA 6.0 by the Neighbor-Joining (NJ) method with 1000 bootstrap replicates. [file Image4.PDF]

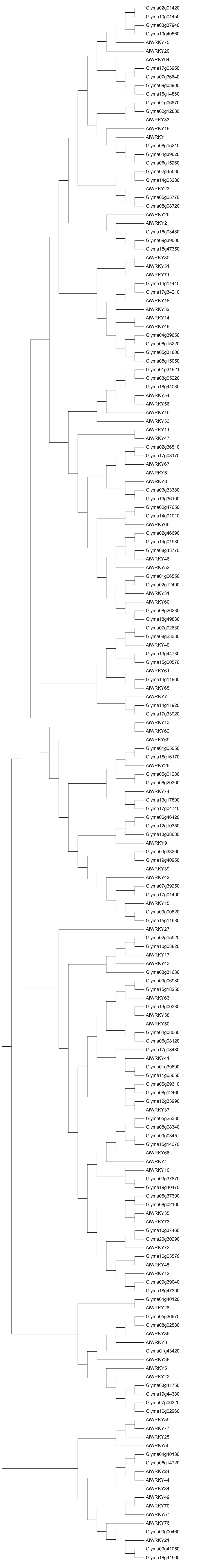

Supplement: Figure S5 — Phylogenetic tree of AiWRKY and GmWRKY proteins. The phylogenetic tree was constructed using MEGA 6.0 by the Neighbor-Joining (NJ) method with 1000 bootstrap replicates. [file Image5.PDF]

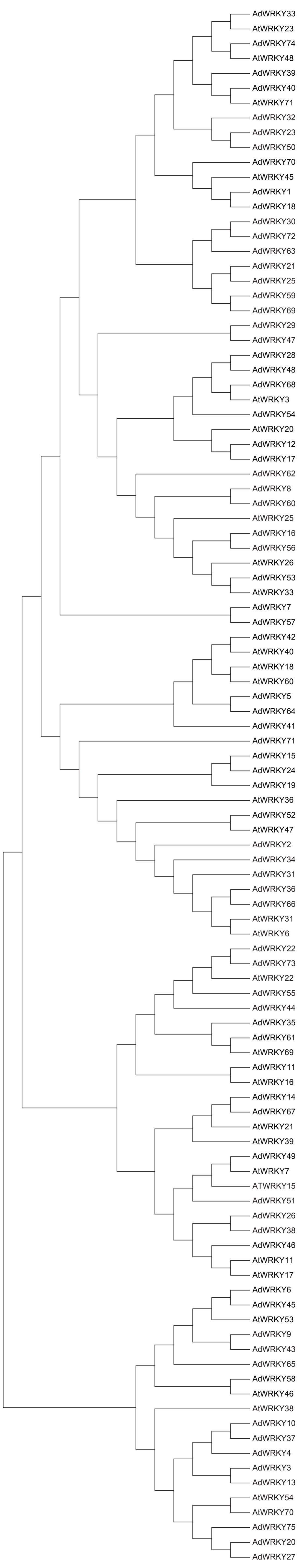

Supplement: Figure S6 — Phylogenetic tree of AtWRKY and AiWRKY genes involved in both response to SA and MeJA. The phylogenetic tree was constructed using MEGA 6.0 by the Neighbor-Joining (NJ) method with 1000 bootstrap replicates. [file Image6.PDF]

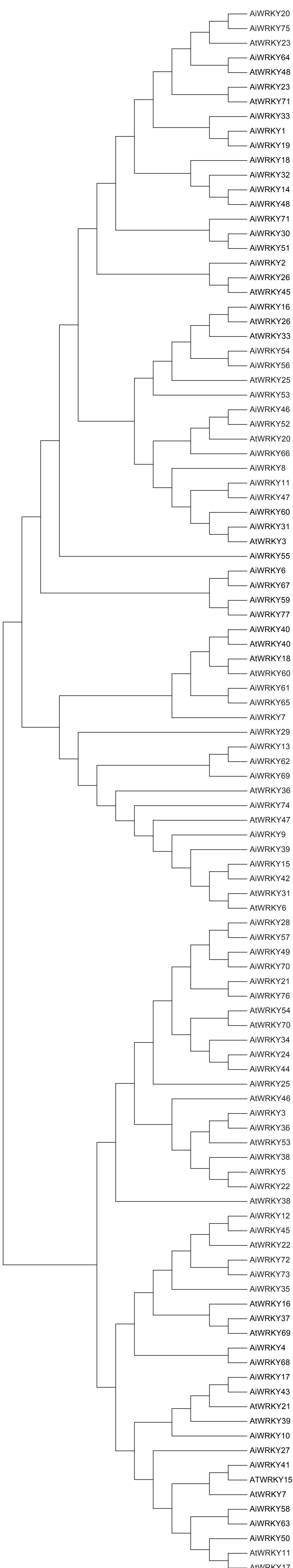

Supplement: Figure S7 — Phylogenetic tree of AtWRKY and AiWRKY genes involved in both response to SA and MeJA. The phylogenetic tree was constructed using MEGA 6.0 by the Neighbor-Joining (NJ) method with 1000 bootstrap replicates. [file Image7.PDF]
